# Evidence-based medicine curricula and barriers for physicians in training: a scoping review

**DOI:** 10.5116/ijme.6097.ccc0

**Published:** 2021-05-28

**Authors:** Alexandra Halalau, Brett Holmes, Andrea Rogers-Snyr, Teodora Donisan, Eric Nielsen, Tiago Lemos Cerqueira, Gordon Guyatt

**Affiliations:** 1 Department of Internal Medicine, Beaumont Hospital, Royal Oak, Michigan, USA; 2 Library Information Services, Beaumont Health, Royal Oak, Michigan, USA; 3 University of Michigan, Ann Arbor, Michigan, USA; 4 Dresden International University, Division of Health Care Sciences Center for Clinical Research and Management Education Dresden, Germany; 5 Department of Health Research Methods, Evidence, and Impact, McMaster University, Hamilton, Ontario, Canada

**Keywords:** Evidence-based medicine, curriculum development, barriers, resident, scoping review

## Abstract

**Objectives:**

To describe the published literature on EBM curricula for physicians in training and barriers during curriculum implementation.

**Methods:**

We performed a systematic search and review of the medical literature on PubMed, Embase, ERIC, Scopus and Web of Science from the earliest available date until September 4, 2019.

**Results:**

We screened 9,042 references and included 29 full-text studies and 14 meeting abstracts. Eighteen studies had moderate validity, and 6 had high validity. The EBM curricular structure proved highly variable in between studies. The majority of the EBM curricula was longitudinal with different lengths. Only five studies reported using Kern's six-step approach for curriculum development. Twenty-one articles reported on EBM skills and knowledge, and only 5/29 full-text articles used a validated assessment tool. Time was the main barrier to EBM curriculum implementation. All the included studies and abstracts, independent of the EBM curriculum structure or evaluation method used, found an improvement in the residents' attitudes and/or EBM skills and knowledge.

**Conclusions:**

The current body of literature available to guide educators in EBM curriculum development is enough to constitute a strong scaffold for developing any EBM curriculum. Given the amount of time and resources needed to develop and implement an EBM curriculum, it is very important to follow the curriculum development steps and use validated assessment tools.

## Introduction

Evidence-based medicine (EBM) has been defined as the "conscientious, explicit, and judicious use of current best evidence in making decisions about the care of individual patients".^1^ EBM involves integrating clinical expertise with the best available evidence in an attempt to bridge the gap between the growing medical literature and patient care while also considering the preferences and values of the patient in the decision-making.^2^ EBM is patient-centered, requires active participation by the learner, acknowledges gaps in knowledge, and bases clinical decisions on evidence rather than authority.^3^

Understanding the concepts of EBM and mastering the abilities to retrieve, critically appraise, and apply medical literature to patient care are essential skills for clinicians.^4^ The Accreditation Council for Graduate Medical Education states that residents must demonstrate the ability to investigate and evaluate their care of patients, appraise and assimilate scientific evidence, and continuously improve patient care based on constant self-evaluation and life-long learning.^5^ Each step of the EBM process requires different levels of competency.^6^^,^^7^ Despite these requirements, most physicians in training have limited knowledge of the EBM process and research methodology, including study design and interpretation of the results.^8^ During the training years, learning fulfills all the adult-learning theory requirements, as the physicians in training come to the table with their own set of life experiences and motivations, they can direct their own learning, they learn better by doing and want to apply their learning to concrete situations sooner rather than later.^9^ As the EBM process is very complex, the learning efficacy is enhanced when it is based on the adult learning theory and when constant, longitudinal exposure pertains over time to the EBM concepts.^10^

Despite the fact that EBM training is now standard in physician training programs, there is still no consensus on how best to ensure that these skills are taught effectively for life-long learning. The most successful teaching methods for EBM are still unknown, and there is little evidence about which teaching methods lead to improved patient outcomes.^11^^-^^16^ It is also extremely important to know of potential barriers that might need to be addressed in order to achieve a successful implementation of the EBM curriculum.^17^^-^^18^

The objectives of this scoping review are 1. Describe the structure of the EBM curricula and its impact on the residents' attitudes, behaviors, skills and knowledge, and 2. Describe the barriers that were identified during the EBM curriculum implementation process.

## Methods

The methodology for this scoping review was based on the framework outlined by Arksey and O'Malley.^19^ The review included the following five key phases: (1) identifying the research question, (2) identifying relevant studies, (3) study selection, (4) charting the data, and (5) collating, summarizing, and reporting the results. The optional 'consultation exercise' of the framework was not conducted.

For the purpose of this scoping review, the word "resident" was used interchangeably with "physician in training", as the term "resident" is mainly used in North America and not widely recognized.  This review was guided by the question, 'What is the structure of the existent EBM curricula and its impact on the residents' attitudes, behaviors, skills and knowledge and what are the barriers identified during the EBM curriculum implementation process? Given the complexity of the research question, we decided that a scoping review would be the ideal study design to answer our question.

### Information sources

The following databases were searched: PubMed, Embase, ERIC, Scopus and Web of Science from the earliest available date for each database until April 25, 2019, and rechecked on September 4, 2019. Gray literature was also searched.  No limits or barriers were applied, but only articles written in English were included. We screened the reference lists of retrieved studies for relevant publications.

### Search and eligibility criteria

PubMed search strategy is described: step #1 "Evidence-Based Practice" [Mesh:NoExp] OR "Evidence-Based Medicine" [Mesh] OR "evidence-based health care" OR "evidence-based healthcare" OR "evidence-based medicine" OR "evidence-based practice" OR "evidence-based care" OR ebm OR ebp OR (critical* AND apprais*) OR ebhc; step #2 "Education, Medical, Graduate" [Mesh] OR gme OR (graduate AND medical AND education) OR postgrad* OR "post-grad" OR "post-graduate" OR "post-graduates" OR residency OR residencies OR resident* OR trainee*; step #3 "Teaching" [Mesh] OR teach OR teaching OR learn* OR curriculum OR curricula OR workshop* OR instruct* OR educat* OR train* OR "journal club" OR "journal clubs" OR lectur* OR module* OR course*; step #4: #1 AND #2 AND #3.

The Appendix contains the search strategies for each database. To identify articles relating to teaching EBM in residencies in curricular format and barriers to teach EBM during the curriculum implementation, the following search terms and their word variants were used: resident, evidence-based, education, teaching, lecture, curriculum, barriers. Exclusion criteria were: student, practitioner, private. The word 'curriculum' was not an inclusion criterion word because not all the articles that described an EBM curriculum had the word 'curriculum' included in the title or abstract. Because the assessment is part of the curriculum development process, studies that reported on an EBM curriculum but didn't have an assessment were excluded. Studies reporting on a single EBM education session or single journal club were excluded as they were thought not to qualify for the curriculum description if they did not address all of the EBM steps.

### Selection of  sources of evidence

We aimed to identify all randomized, nonrandomized, and before and after studies that reported on: 1. teaching EBM in a curriculum format to residents and on: 2. barriers during curriculum implementation. To be included, studies had to provide details about the format of their EBM curriculum and report on the evaluation of their curricula, as evaluation is the last step in the curriculum development process. We used the following criteria to screen studies for inclusion in our review: 1. Study design: original studies and abstracts presented at national conferences, quantitative, qualitative, and mixed methods studies were included if they reported on EBM curriculum with or without barriers to teaching EBM to residents; abstracts describing an EBM curriculum and its evaluation were included to avoid the risk of reporting bias if they had been excluded. Systematic reviews on the EBM curriculum and barriers were not included. Their references were manually verified against our search, and the articles not initially included were uploaded for screening; 2. Population: residents of any specialty, working under the supervision of a medical specialist. Medical students and attending physicians were not included as their learning environment, patient care exposure, and motivation are significantly different than for the physicians in training; 3. Outcomes: pertaining to curriculum evaluation, the following categories based on Kirkpatrick's training evaluation model^20^ were used to assign the types of outcomes for included studies related to teaching EBM to residents: level 1 (attitudes toward EBM and participants' satisfaction), level 2 (EBM skills and knowledge), level 3 (behavior, the degree to which participants apply what they learned during training) and level 4 (results, the degree to which targeted outcomes occur as a result of the training, patient-related outcomes); barriers reported during curriculum implementation were collected, and categorization was reported.

### Data charting process

A detailed excel spreadsheet with all the data points that were planned to be collected was developed through collaboration between 2 of the authors (AH and BH). The form was calibrated through concomitant (both authors) analysis and data extraction from various studies. Data charting was done independently and in duplicate. Potentially relevant article abstracts were independently reviewed by two authors (AH and BH), who then met and agreed on article selection. For each potentially eligible study, the full paper was read and reviewed by the same two authors (AH and BH) independently. They both assessed whether the study fulfilled the inclusion criteria. Studies about which both reviewers had doubt or disagreement about inclusion were thoroughly discussed during three consensus meetings, and a common decision was reached.

Two authors (TD) and (BH) performed the data extractions from the full-text articles included, which were then fully reviewed by another author (AH) and any disagreements on data extractions were resolved by consensus between AH and BH. Two authors (BH) and (EN) performed the data extraction from the abstracts included. A prespecified data extraction form was used to extract and collect information from the included studies on the author, year of publication, type of study design, country, residency type, number of participants, study period, specific details of the EBM curriculum structure, length, teachers, content, outcome measures, evaluation methods, results, and barriers. Because we expected to find no homogenous studies in this area, no pooling of data was attempted.

### Critical appraisal of individual sources of evidence

To assess the risk of bias of individual studies, two authors (BH and TD) independently rated the quality of the studies at the study level. All disagreements were reviewed and resolved by a third author, AH.

### Risk of bias assessment of individual studies

We assessed the quality of the pre-post studies using the National Heart, Lung and Blood Institute Quality Assessment Tool for Before-After (Pre-Post) Studies with no Control Group (Table 1).^21^ Maximum points for each study were 12. We added scores for each criterion together and divided them by 12. The risk of bias rating was: low (75-100%), moderate (25-75%), or high (0-25%). For the randomized controlled trials, quality was assessed according to the Cochrane Collaboration recommended methodology.^22^ Each criterion was assessed as high, low, or unclear risk of bias. The quality of cohort studies included in this review were assessed using the Newcastle-Ottawa Scale (NOS),^23^ which assigns a maximum of 9 points to each study. The assessment scale analyzes three broad perspectives of each study: the selection of the study groups, the comparability of the groups, and the ascertainment of either the exposure or outcome of interest for cohort studies. When a study received 3 or 4 pluses in the selection domain and 1 or 2 pluses in the comparability model and 2 or 3 pluses in the outcome/exposure domain, it was considered to be of good quality (or low risk of bias); if the study received 2 pluses in selection domain and 1 or 2 pluses in comparability domain and 2 or 3 pluses in outcome/exposure domain, it was considered of moderate quality; if a study received 0 or 1 plus in selection domain or 0 pluses in comparability domain or 0 or 1 plus in outcome/exposure domain, it was deemed to be of high risk of bias (Table 1).

## Results

### Selection of sources of evidence

Nine-thousand-forty-two references were imported for screening, and 214 duplicates were removed. Eighteen extra articles were considered for inclusion from a manual review of the references, and after cross-checking with the articles already included in the initial screening, 8 of them were duplicates and were removed. Four extra articles were suggested by experts in the field and were included for screening. A total of 9064 articles were included after automatically removing the duplicates. Further duplicates were removed manually, and 8842 articles were screened against the title and abstract, and 8742 articles were found irrelevant and were excluded. One hundred articles were assessed for full-text eligibility, and 57 articles were excluded because they didn't meet the eligibility criteria. Only one out of the 18 studies manually added after searching the references was added to this review. One of the 4 studies suggested by the experts fulfilled all inclusion criteria. The final review included 43 articles (29 full-text articles and 14 abstracts) describing the structure of the EBM curriculum. Thirteen of these articles reported on barriers to EBM curriculum implementation.

**Table 1:** Criteria for assessing the risk of bias of studies included

Pre-Post study Design^21^	
	1. Was the study question or objective clearly stated?
	2. Were eligibility/selection criteria for the study population prespecified and clearly described?
	3. Were the participants in the study representative of those who would be eligible for the test/service/intervention in the general or clinical population of interest?
	4. Were all eligible participants that met the prespecified entry criteria enrolled?
	5. Was the sample size sufficiently large to provide confidence in the findings?
	6. Was the test/service/intervention clearly described and delivered consistently across the study population?
	7. Were the outcome measures prespecified, clearly defined, valid, reliable, and assessed consistently across all study participants?
	8. Were the people assessing the outcomes blinded to the participants' exposures/interventions?
	9. Was the loss to follow-up after baseline 20% or less? Were those lost to follow-up accounted for in the analysis?
	10. Did the statistical methods examine changes in outcome measures from before to after the intervention? Were statistical tests done that provided p values for the pre-to-post changes?
	11. Were outcome measures of interest taken multiple times before the intervention and multiple times after the intervention (i.e., did they use an interrupted time-series design)?
	12. If the intervention was conducted at a group level (e.g., a whole hospital, a community, etc.) did the statistical analysis take into account the use of individual-level data to determine effects at the group level?
Prospective cohort study design^23^	
Selection	
	1) Representativeness of the exposed cohort
	2) Selection of the non-exposed cohort
	3) Ascertainment of exposure
	4) Demonstration that outcome of interest was not present at the start of the study
Comparability	
	1) Comparability of cohorts on the basis of the design or analysis controlled for confounders
Outcome	
	1) Assessment of outcome
	2) Was follow-up long enough for outcomes to occur
	3) Adequacy of follow-up of cohorts
Randomized controlled trial study design^22^	
	1) Random sequence generation
	2) Allocation concealment
	3) Selective reporting
	4) Other sources of bias
	5) Blinding (participants and personnel)
	6) Blinding (outcome assessment)
	7) Incomplete outcome

### Characteristics of sources of evidence

Of the 43 studies included in the final systematic review, 29 studies were reported as full text as follows: 18 described the results of quantitative pre-post study design, 5 described the results of quantitative surveys, 4 described prospective cohort studies, two randomized controlled trials, and 14 meeting abstracts. Table 2 and Table 3 present a more detailed description of the studies. Most studies included were performed in countries where English is the primary language: 40 in the United States, 2 in Canada, and one in the Netherlands. Full-text articles included between 4 and 181 residents, and the abstracts included between 11 and 108 residents. One full-text article and two abstracts didn't report on the number of residents enrolled. The specialties included were varied; the majority were internal medicine (12 full studies and ten abstracts). Other specialties included were: emergency medicine (3 full studies^15^^,^^16^^,^^17^ and ^2^ abstracts^24^^,^^25^), family medicine (7 full text studies), pediatrics (4 full text studies^4^^,^^12^^,^^17^^,^^18^ and 1 abstract^26^), 1 neurology^27^^, ^^1^ general surgery^28^ and ^2^ obstetrics and gynecology^29^^,^^30^ full studies. One abstract didn't specify the residency type.^31^ The study period reported was as short as six weeks^32^ and as long as five years.^32^^,^^33^ One study was a single point in time survey.^27^ One full-text study^34^ and 3 abstracts^35^^-^^37^ didn't report on the study period.

#### Critical appraisal within sources of evidence

The risk of bias was assessed only for the studies included as full text. The abstracts were not assessed for their quality, as it was appreciated that they contained very limited information about the methodology and possible bias. Also, the full-text surveys were not assessed for risk of bias. The overall quality of the 24 studies evaluated was determined to be moderate or low risk of bias (Appendix 1 and Appendix 2).

**Table 2:** Description of studies included in the review of research on residents' evidence-based medicine curricula

Author (year)	Allan et al. (2008)	Aneese et al. (2019)		Bentley et al. (2018)		Burneo et al. (2006)	Chitkara et al. (2016)	Friedman et al. (2010)
Study design	Pre-post study	Pre-post study		Pre-post study		Survey	Pre-post study	Pre-post study
Country	Canada	USA		USA		Canada	USA	USA
Residency type	Family medicine	Internal Medicine		Emergency Medicine		Neurology	Pediatrics	Emergency Medicine
Number of Participants, n	181	60		53		12	17	24 seniors (PGY-3 and PGY-4)
Study period	3 years	1 year		1 year		single point in time survey	3 years	3 months
	
EBM Curriculum Structure	Workshop, Family Medicine Desktop, brief evidence- based assessment of the research [BEAR], journal club; teaching on industry–physician interaction, presentation skills, and a quarterly review of the literature	1)Monthly EBM workshop; 2)monthly Journal Club; 3)resident led morning report; 4)teaching rounds		8 self-paced modules		24 clinical topics chosen at beginning of the year; prior to each session, resident chooses real encounter on the topic, develops question, searches, selects best article and distributes to all trainees and faculty. CAT is designed and published to website.	1) first year residents: foundational course as small groups and independent study 2) 2nd year: application, CAT presentation 3) 3rd year: integration (act as mentors to 1st and 2nd)	1 hour EBM online tutorial; 3 dedicated formal literature searches for active management questions; brief EBM teaching round (reviewed the clinical question, method, literature search and the relevant evidence was used to answer the management question)
Length/frequency	1) Workshop: 4 half-days, each consisting of a one-hour lecture followed by a two-hour, small- group session. 2) Each BEAR is presented in approximately 15 minutes; 3- 4/resident; 3) Journal club - 2.5 hours	1) 90 minutes X 4/year; 2)60 minutes X 12/year; 3)1 hour X 1/week; 4)1 hour X 3/week		Monthly		Biweekly (every other week), 90 minutes (total of 24 per year)	Longitudinal, year long, frequency not specified	1 hour tutorial; 3 x 8 hour shifts
Teachers/ facilitators	Faculty physician, faculty librarian, second-year resident	Faculty physician, faculty librarian, second and third year resident		Not reported		Faculty physicians, residents of all levels	Faculty, librarian, third year residents as mentors	Not reported
Clinically integrated (lectures versus point of care)	Yes, partially	Yes, partially		No		No	Yes, partially	Yes
	
Curriculum content	Knowledge component: 1) Understand the rationale and benefits of EBM 2) Provide a strong foundation in the basic principles of EBM, including: Recognizing and formulating clinical questions; Finding and accessing information, interpreting information, applying information 3) Provide and identify online resources, educational tools, and web-links. Skill component: 1) Learn to identify problems/questions encountered in practice and seek solutions by rapidly answering clinical questions with best evidence 2) Build skills and comfort in knowledge transfer. Attitude component: 1) Facilitate appreciation and enthusiasm for the judicious use of current evidence to optimize patient care, and Maintenance of skills and knowledge through life-long self-directed learning.	Formulating clinical question; Search skills; Critical appraisal skills; Evaluating the evidence skills; Applying the evidence		Critical appraisal skills; EBM concepts based on study design		Formulating clinical question, finding evidence, critical appraisal	1) PICO, search skills, critical appraisal 2) PICO applied, CAT presentation, CAT abstract generation 3) EBM teaching and mentorship	PICO, Formulating clinical question; Search skills; Critical appraisal skills; Evaluating the evidence skills; Applying the evidence
Curriculum development process described	Yes, quite extensive. Comittee formed to investigate concerns regarding previous curriculum, goals and objectives developed	Not discussed		Not discussed		Not discussed	Not discussed	Yes, Kerns six step approach used
	
Outcome measures	Self-assessed skills, past training, and objectives in EBM	EBM knowledge and skills; Attitude toward EBM		EBM knowledge and skills; attitude toward EBM		Participation during EBCP sessions, applicability to daily clinical practice, and foreseeable future practice, how likely clinical practice was influenced, if trainees were teaching the concepts to other residents/students	a. Knowledge and skills b. Attitudes and behavior	Practice norms and attitudes regarding EBM; knowledge, skills

Evaluation method	pre-curriculum survey, quiz; post survey, quiz	Validated questionnaire (Fresno test); Questionnaire; Focus group		validated questionnaire (Berlin Questionnaire BQ)		questionnaire/survey	a. Pre-post knowledge questionnaire (1st year), b. self assessment survey (years 2 and 3), c. focus groups	survey
						
Results	Statistically significant improvement in all areas of comfort with evidence-based practice and opinions regarding the curriculum; general opinion (e.g., The practice of EBM results in better patient care) showed improvement as well, albeit to a lesser degree;	Fresno pre-test 110.5, post-test 115 (p=0. 60); pre-post therapy questionnaire (p=0. 006), diagnosis (p=0.006), systematic review (p=0.002), harm (p=0.004)		BQ median pre scores 40%, median post scores 73.3% (p=0.002)		On a scale of 1 to 10, they rated the influence to include EBCP concepts in their daily clinical practice as high (mean 6.8, S.D. 1.5). All graduates believed the EBCP concepts were useful, but only applied them when time allowed. On a 1-to-10 scale, they rated the influence to include EBCP concepts in their daily clinical practice higher than the residents (mean 8.5, S. D. 1.2).	pre 6.9, post 9.1 (p=0.02); institutional practice changes reported, such as creating new care plans; residents were better able to appraise the literature (p=0. 02); improved reported searching skills, appraisal, clinical application, repeated practice in the peer mentor role improved knowledge and skills retention	No significant change in the number of subjects that anticipated further practice changes as a result of their experience; 16.3% change in management by the primary team based on the literature searches performed; the change in management was a perceived change as reported by the subjects and was not reviewed by an attending physician.
	
Author (year)	Gehlbach et al. (1980)		George et al. (2012)	Green and Ellis (1997)		Halalau et al. (2016)	Keddis et al. (2011)	Kenefick et al. (2013)
Study design	Prospective cohort		Pre-post study	Pre-post study		Pre-post study	Prospective cohort	Pre-post study
Country	USA		USA	USA		USA	USA	USA
Residency type	Family Medicine		Family Medicine	Internal Medicine		Internal Medicine	Internal Medicine	Pediatrics
Number of Participants, n	23 PGY-2 and PGY-3		26 PGY-2	34 (PGY-2 and PGY-3)		23	45 PGY-3, 42 PGY-1	4 x PGY-1
Study period	2 years		2 years	1 year		1 year	5 months	1 month

EBM Curriculum Structure	Seminar sessions, resident presentations		One-on-one 1-hour monthly meetings	Based on adult learning theory, the educational strategy included a resident- directed tutorial format, use of real clinical encounters, and specific EBM facilitating techniques for faculty		Based on adult learning theory; resident-directed teaching, small-group sessions, open discussion sessions and direct one-on-one teaching of EBM concept	PGY-1 residents participate in 8 interactive 1-hour sessions during a 1-month rotation. Residents apply their knowledge by presenting their EBM analysis of a clinical case and pertinent literature to a group of residents and faculty members at a 1-hour weekly conference called Clinical Decision Making Journal Club	2 sessions
Length/frequency	8 x 1 hr weelky sessions		1h monthly meetings (30 min/ meeting reserved for EBM) x 1 year	7-week, weekly 1 h tutorials, for 5-14 residents		Over 10 hours of EBM training each month, for 12 months	8 x 1 hour sessions	2 x 2h sessions during a month
Teachers/ facilitators	Faculty		Learning coach = family physician and recent residency graduate	General medicine faculty, librarian		Faculty, librarians, residents	Not reported	Medical librarian and hospitalist
Clinically integrated (lectures versus point of care)	No		No	Yes		Yes, partially	No	Yes

Curriculum content	First 3 seminars: clinical trials, sensitivity, specificity, predictive value, statistical significance; journal handouts for each session; Next 4 seminars: resident presentations evaluating articles; Last seminar: take-home test		EBM curriculum topics: Introduction to EBM, introduction to online EBM resources, introduction to PubMed, basic biostatistics, Introduction to guidelines, formulating PICO Questions, study design, clinical question search strategies	Each of the seven tutorials guides the residents through a real clinical scenario, representative of one of six prototypical clinical questions: therapy, prognosis, harm, diagnosis, prevention, and decision making. In the seventh, they can choose any question type, but must use a systematic review as evidence.		1) Fundamentals of EBM and their application to patient care; study designs and simple statistics and their interpretation in patient care; 2) sessions on using and searching EBM resources; learn the hierarchy of evidence and to gain searching skills in finding the best evidence to inform decision making for patient care; 3) skills in critical appraisal of any study design; 4) critical appraisal of a study that would answer their clinical question and receive direct peer to peer and EBM faculty feedback; 5) basic study design and statistical analysis interpretation of therapy studies, diagnosis studies, harm studies and systematic reviews and meta- analysis	Sessions address the following topics: the EBM cycle; the patient, intervention, comparison, outcome, type of study design, type of question (PICOTT) format for structuring clinical questions; hierarchy of evidence; study design; and critical appraisal of therapy, diagnosis, prognosis, meta-analysis, and harm articles.	PICO format to help form and organize a clinical question; search of the literature to answer their PICO question, levels of evidence, optimal study types
Curriculum development process described	Not discussed		Yes, minimal components included (needs assessment, implementation, evaluation)	Yes, most components (needs assessment, goals and objectives, implementation, assessment)		Yes, fairly robust (questionable needs assessment. Yes implementation, assessment)	Not discussed, pre-existing curriculum	Not discussed

Outcome measures	Skills to interpret medical literature critically		Attitudes regarding EBM; EBM knowledge and skills	Attitudes toward EBM, prior critical appraisal training, self-assessed EBM competence, medical reading habits, and preferences for information sources.		Residents' comfort level with basic statistics, literature search and EBM concepts, residents' satisfaction with the EBM curriculum	Resident confidence with and knowledge of EBM topics	Scores on in-class, independent searches, and posttest cases, each of which were scored independently by three faculty members

Evaluation method	take-home test w multiple choice questions centered around some article samples; a practical problem given to all residents at the in training exam; comparison between 23 PGY 2-3 who took curriculum and 12 PGY-1 who did not (for the in training exam problem)		attitudes - survey; knowledge - 18 item quiz, 6 questions adapted from Fresno test of competence; qualitative - coach ratings after every coaching session	survey of EBM behaviors, a survey of self- assessed EBM competences, and an EBM skills test - modified from Stern et al. (free text responses to questions based on a clinical vignette and a redacted journal article)		27 item survey	Likert- scale type survey (which included questions from the validated Berlin questionnaire)	pre-test and posttest of four cases to evaluate the residents' skill and efficiency in asking a clinical question and finding an appropriate answer
						
Results	Participants' % scores on the article analysis questions ranged from 42-93%; nonparticipants scored from 34-100%. Participants had a mean score of 73.8 %, nonparticipants had a mean 63.8 % (stat signif). When median percent scores were compared, a larger difference was observed between the resident groups (79 participants, 62 nonparticipants)		Favorable attitudes; significant improvement in quiz scores by 31.8% (p<0.001); using wider range of electronic resources; greater confidence and comfort in finding clinical information; ability to find best medical evidence for patients in real time / point of care; EBM valued; importance of one-on-one format	Scores on the EBM skills test (8.5 to 11.0, p =.001) versus control (8.5 to 7.1, p=0. 09). Posttest scores for case and control subjects, the mean difference was 3.9 points (p =.001, 95% confidence interval [CI] 1.9, 5.9).		Self-reported conceptual understanding improved for: relative risk 14%, odds ratio 14%, confidence intervals 27%, and number needed to treat 12%. Comfort with meta-analysis appraisal improved, from 30% to 38%. Routine appraisal sheet use increased by 31%. A 17% increase in satisfaction with the EBM curriculum was reported.	There was no relationship between confidence with and actual knowledge of EBM topics (PGY-1 pre-curriculum vs PGY- 3). Lower confidence among PGY-3 than among PGY-1 internal medicine residents for several EBM topics. PGY-3 residents demonstrated poor knowledge of several core topics taught during internship.	PICO: pre-curriculum test score 29%, post- curriculum test score 87%; Searching: Pre- curriculum test scores 46%, post- curriculum 99%; average time to complete cases pre-curriculum 12 min, versus post-curriculum 8.10 min.
	
Author (year)	Kim et al. (2008)	Kitchens and Pfeifer (1989)	Kohlwes et al. (2006)		Konen and Fromm (1990)	Kortekaas et al. (2016)	Lentscher and Batig (2017)
Study design	RCT	Pre-post study	Prospective cohort		Survey	RCT	Pre-post study
Country	USA	USA	USA		USA	Netherlands	USA
Residency type	Internal Medicine	Internal Medicine	Internal Medicine		Family Medicine	General Practice	Ob/Gyn
Number of Participants, n	50 (PGY-2 and PGY-3)	83 (PGY-1, PGY-2, PGY-3)	32 PRIME residents		12	79	17
Study period	1 year	unclear	2001-2004		5 years (1983-1988)	2 years	1 year

EBM Curriculum Structure	EBM teaching workshops (intervention) vs weekly resident journal clubs without a formal or explicit curriculum (control)	Group A - literature-based curriculum in clinical appraisal; Group B - ambulatory care medicine topics	4 components: (1) didactic lecture, (2) frequent journal clubs, (3) work-in-progress sessions, and active mentoring to enable residents to "try out" a clinical research project during residency, (4) research project supervision		PGY-1: 2 seminars, a journal club, quarterly research gatherings; PGY-2: presentation at grand rounds; PGY-3: seminars	Intervention: integrated EBM training and teaching sessions based on dilemmas from actual patient consultations; Comparison: stand-alone EBM training at the institute only	Structured journal club curriculum
Length/frequency	6 x 2h EBM teaching workshops during a month	weekly preclinic conference 30-45 min; 17 weeks for Group A, 8 weeks for group B	(1) 12 didactic lectures in consecutive weekly 90- minute sessions; (2) weekly afternoon small journal clubs; (3) PRIME projects - residents present their projects quarterly in 90 min work-in-progress sessions (4) research project supervision: 1-2 x 1 h meetings/month		3 year curriculum; PGY-1: 2 seminars and a journal club, quarterly research gatherings (length not clear); PGY-2: 1 hour presentation at grand rounds; PGY-3: monthly 2 h seminars	a) Clinical practice: 1 h x 4 days/week; integration of EBM 1/week; critical appraisal 1/month b) institute: 1 day/week; EBM course 5 days/year, 2.5 hours; exchange of last week's experiences in clinical practice 1 h/week, integration of EBM for 15 min	Monthly
Teachers/ facilitators	Medical librarians, resident-led	Faculty preceptors	Members of clinical investigator faculty, program director, program directors, clinician-investigator faculty, faculty		Faculty members, physician research director, nonphysician biostatistician, research assistant	Experienced GPs	Not reported
Clinically integrated (lectures versus point of care)	Yes	No	No		No	Yes, partially	No

Curriculum content	Formulating clinical questions using PICO; medical librarians tought evidence-based synopses and summary resources; topics of therapy, prevention, diagnosis, and prognosis - led by a resident who identified questions from actual patient encounters and performed literature searches using both bibliographic databases as well as evidence-based summary resources to find articles that addressed their question	Series of articles published by the Department of Clinical Epidemiology and Biostatistics at McMaster University that provides a reader with practical guidelines to help read the clinical literature critically. Each week one of these articles was paired with a current article from the clinical literature. Each resident was expected to read both articles, and one resident was assigned to lead a discussion of the content of the clinical article as well as the methodological strengths and weaknesses of that article	(1) PRIME epidemiology curriculum: A. overview of epidemiology study types, B. How epidemiology data are presented; C. How to design a research question; D. Study design and sampling; E. Issues in measurement; F. Causal inference; G, H. Qualitative biostatistics; I. Computer skills; J. Research ethics; K. Systematic review; L. Meta-analysis (2) Weekly journal clubs: diagnostic test evaluation, case- control studies, cohort studies, randomized control trials, meta-analysis, decision analysis, cost- effectiveness analysis, practice guidelines, and clinical overviews are covered, and articles that they frequently hear quoted on the medical wards.		1) Critically evaluate research studies in the professional literature and translate valid conclusions into medical practice; 2) identify areas of potential research interest; 3) embark on a lifelong program of continuing education and professional growth.	Integration of EBM, critical appraisal of an article, EBM course focusing on translation of evidence into clinical practice, patient-related preassignments and postassignments to perform with supervisor; exchange of last week's experiences in clinical practice; discussion of barriers; presentation of a critically appraised topic; possibility for e-learning, online coaching, participation in research	Monthly curriculum that paired a chapter/topic from 2006 edition of The Lancet Handbook of Essential Concepts in Clinical Research with a contemporary journal article selected to highlight the month's topic: overview of clinical research, descriptive studies, bias and causal associations, cohort studies, case-control studies, finding controls for case-control studies, overview of clinical research, uses and abuses of screening tests, refining clinical diagnosis with likelihood ratios, sample size calculations in RCTs, general allocation sequences in RCT, unequal group sizes in RCT
Curriculum development process described	Not discussed	Not discussed	Not discussed		Not discussed	Not discussed	Not discussed

Outcome measures	EBM knowledge, use of evidence-based resources, performance on web-based clinical vignettes	Basic knowledge of clinical epidemiology	Clinical competence, board passing rates, asked to be chiefs, PRIME program popularity, research output		Attendance (as the best measure of interest); resident ratings (attitudes) of each session	EBM behavior, assessed by measuring guideline adherence (incorporating rational, motivated deviation) and information-seeking behavior; EBM attitude, EBM knowledge	Attitudes and self-rated knowledge; EBM knowledge

Evaluation method	Fresno test pre- and post-intervention. Post intervention, residents twice completed a web-based, multiple-choice instrument (15 items) comprised of clinical vignettes, first without then with access to electronic resources.	clinical epidemiology test 1 month post- intervention for group A and group B; intervention changed for group A and group B (cross over); then test again; test with 22 questions on clinical epidemiology principles derived from the articles by the McMaster University group	clinical competence scores		pre-test taken from Reigelman	1. EBM behavior: information-seeking behavior - logbooks; guideline adherence - validated instrument; 2. EBM attitude: McColl validated questionnaire; 3. EBM knowledge: MF Kortekaas validated questionnaire	survey: 6 initial questions to assess individual attitudes and self-rated knowledge; additional questions to assess individual knowledge related to basic study design, bias, and levels of medical evidence. administered pre- and post-intervention
						
Results	Posttest scores improved for both groups, but were significantly higher for the EBM teaching group (mean score increase 22 (SD=13.8) for teaching group vs. 12 (SD= 12.2) for control group, p=0. 012). There was more improvement in EBM knowledge (100-point scale) for the intervention group compared to the control group (mean score increase 22 vs. 12, p=0.012).	Group B performed significantly better on the second test (post) than on the first test (pre), 68.5% vs 63.3% (p=0.034), while group A did not improve (64.5% va 65.9% - group who received the intervention from the beginning).	The overall clinical competence scores evaluating the 32 PRIME residents gave an overall average score of 8.23 on a 9.0 point scale. This was significantly better than the average of 8.09 for the rest of the internal medicine program (p<0.001).		Attendance greatly improved after the first year (p<0.001); seminar topic preferences (computers in medical practice 0.99; how to write a research article 0.98; different study designs 0.91; reading a research article 0.88; biostatistics 0.86; conducting a literature review 0.75; clinical trials 0.71)	No significant differences in outcomes between the 2 groups, with relative risks for guideline adherence varying between 0.96 and 0.99 (95% CI 0.86 to 1.11) at the end of the third year, and 0.99 and 1.10 (95% CI 0.92 to 1.25) at 1 year after graduation, and for information-seeking behaviour between 0.97 and 1.16 (95% CI 0.70 to 1.91) and 0.90 and 1.10 (95% CI 0.70 to 1.32), respectively.	There was no significant improvement in resident self-assessed knowledge following curriculum implementation. There was a trend toward improved objective knowledge pertaining to study design and interpretation after curriculum completion, but this was not statistically significant
Author (year)	Letterie and Morgenstern (2000)	Luciano et al. (2016)	Mohr et al. (2015)		Nelson et al. (2017)	Nicholson and Shieh (2005)	Ross and Verdieck (2003)
	
Study design	Survey	Prospective cohort	Pre-post study		Pre-post study	Survey	Pre-post study
Country	USA	USA	USA		USA	USA	USA
Residency type	Ob/Gyn	Internal Medicine	Emergency Medicine		Pediatrics (PGY-1 and PGY-2)	Internal Medicine	Family medicine
Number of Participants, n	not provided	61	31		60	36	18 - intervention; 30 - control
Study period	1 year	2 months	2 years		1 year	1 year	1 year

EBM Curriculum Structure	Journal club: 2 sets of articles distributed for each session; 1 - literature on topics of epidemiology, biostats, experimental design (2-6 articles); 2 - clinical literature for review (illustrate and emphasize prior concepts)	Patient-centered ambulatory morning report (AMR), which combines the User's Guides to the Medical Literature approach to EBM and the Kolb experiential learning theory	Journal club		Sessions grounded in adult learning theory principles, flaw catching exercises, multiple choice questions using audience responses and interactive review of abstracts and articles	Daily EBM session during rounds with the hospitalist team	Interactive workshops
Length/frequency	One 2h session/ month x 12 months	Morning report: 2-3 months/year, weekdays, 30 min/day; + 5h basic EBM concepts; + 4h interactive didactic EBM content (most of these 9h take place within morning report)	monthly		4 x 90 min sessions	1 month	10 sessions; 1-2 hour sessions, brief lecture (30- 40 min) followed by practical application
Teachers/ facilitators	Resident, staff physician	Residents, facilitators (not specified)	Faculty		Faculty	Hospitalist	Preceptors
Clinically integrated (lectures versus point of care)	No	No	No		No	Yes	Yes

Curriculum content	24 concepts descriptive of topics considered critical in the assessment and evaluation of literature descriptive of clinical practice and patient care: topics of epidemiology, biostatistics, and experimental design (p values -> meta analysis), literature on clinical topics (illustrate and emphasize concepts of experimental design and statistical analysis).	EBM skills: identify a clinical problem, define the problem as a structured clinical question, acquire new knowledge; EBM competencies: evaluate the validity of evidence cited, evaluate the results; apply and use new knowledge; EBM competency: apply the results to the clinical scenario	1) Dedicated 20-min monthly discussion led by a faculty member during monthly journal club focused on an EBM-focused topic. 2) a single faculty member being grouped with three coordinating residents to review selected articles from an EBM perspective prior to journal club to discuss the teaching points of each in detail. 3) a period of peer-to-peer teaching during journal club, 4) a dedicated core of EBM faculty dedicated to directing the journal club curriculum and establishing expertise in EBM concepts		Session 1: class - EBM test, flaw catching exercise, Introduction to MAARIE framework; homework - multiple choice questions covering session 1 information; Session 2: class - review multiple choice questions in teams using audience response systems; introduction to MAARIE framework; flaw catching exercise; homework - read randomized controlled trial; Session 3: multiple choice questions covering session 2, apply MAARIE framework to abstracts; apply MAARIE framework to RCT #1; homework - read RCT #2; Session 4: review MAARIE framework and key concepts; apply MAARIE framework to RCT #2, EBM test.	Definitions and principles of EBM, literature appraisal, EBM internet resources; formulate focused clinical questions for each patient admitted, do literature searches, use EBM principles to evaluate search results, and present findings during subsequent rounds.	1. Multiple-choice pretest, overview and constructing focused questions: interactive formulation of good, researchable EBM questions for patient care; 2. categorizing articles, reading review articles: discussion of recognizing focused reviews, how to sort articles into groups (i.e., therapy, diagnostic tests, reviews); 3. reading review articles: a discussion regarding key features of well-researched and written reviews; 4. evidence for therapy; 5. evidence for diagnostic tests; 6. evidence for history and physical exam; 7. evidence for prevention; 8. evidence for practice guidelines; 9. applying quality improvement
Curriculum development process described	Not discussed	Not discussed	Not discussed		Not discussed	Yes, everything but discussing needs assessment	Yes, Kerns six step approach used

Outcome measures	EBM knowledge and skills	EBM knowledge: learn to use medical literature to guide patient care	EBM skills and knowledge		Baseline knowledge, efficacy of the curriculum in improving knowledge	Learning experience, understanding of EBM terms or practice skills	Knowledge and application of EBM during continuity clinic patient care

Evaluation method	questionnaire	18-question EBM test (of the 18 questions, 1 required formulating a structured clinical question, 6 addressed evaluating validity of an article, 5 addressed evaluating results, and 6 addressed applying results to a clinical scenario).	Fresno test		validated 20 question evidence based medicine multiple choice test was administered on three separate occasions	questionnaire on which they were asked to rate the impact of the curriculum on their understanding of 20 EBM terms or practice skills	a 50-item, multiple-choice examination was administered before and after the workshop series; residents at another FP residency at the same university served as a control group. Resident–preceptor interactions during outpatient continuity clinic were tape recorded prior to and six months following introduction of the curriculum
						
Results	85% residents wanted to continue the format without much change; 15% wanted to restrict literature to Ob/Gyn content	Average score increased during the training period, most strongly following internship. Mean scores stabilized after internship; however, the range of scores successively narrowed; EBM scores improved in time in the following domains: formulating a structured clinical question, assessing the validity, and evaluating the results	Total test scores did not increase significantly (105.4 vs. 120.9, p = 0.058) in the before–after analysis; the only subscore showing improvement was interpretation of study validity (32.1 vs. 40.4 points, p = 0.03); attendance was significantly associated with Fresno test score, with those attending more than 6/11 sessions (55%) scoring 28.2 points higher (p = 0.003)		Post curriculum, the fall group’s scores improved 23% from baseline (M=10.3, SD=2.4) to (M=12.7, SD=3.0) students (t(26)=-3.29, p=0.0018) while the spring group improved by 41% (M=10.0, SD=2.8) to (M=14.1, SD=2.2) students (t(32)=- 6.46, p<0.0001). There was an association between number of sessions attended and increase in post curriculum score (χ2 (3, N=60) =11.75, p=0.0083).	Results were very positive with average effect of more than 4 (somewhat strong effect/impact) for 16 of the 20 questions.	Pre-intervention multiple-choice test results were similar (control mean 56%, experimental 53%, p>. 22 NS); post-intervention test scores for the experimental group were significantly improved (mean 72%, p<.001); there was no significant improvement in test results among members of the control group (p>.05 NS); in the recorded resident–preceptor interactions, a marked increase in the use of EBM terms indicated awareness and/or use of EBM in the experimental group

Author (year)	Shaughnessy et al. (2012)	Thom et al. (2004)	Trickey et al. (2014)	Windish (2011)	Zeblisky et al. (2015)
Study design	Pre-post study	Pre-post study	Pre-post study	Pre-post study	Survey
Country	USA	USA	USA	USA	USA
Residency type	Family Medicine	Family Medicine	General surgery	Internal medicine	Pediatrics
Number of Participants, n	23	13 PGY-1	40	52	46
Study period	5 years	3 months	6 years	2 years	6 months

EBM Curriculum Structure	1) Block format - intensive instruction over the course of 1 month (30 hours); 2) longitudinal series of ongoing conferences: a modified journal club, group session at the beginning of the year, weekly subspecialist conferences; teaching was also integrated into day-to- day clinical activities via precepting interactions	Individual EBM rotation for interns; online tutorial, workshop, journal club	Lectures, tutorials, practice questions, examples	Journal articles reading, seminars, presentations by learners	1) Technology sessions, 2) increased librarian support for EBM presenters, 3) increased interaction during conferences, 4) standardized curriculum to reflect progression of the academic year, and 5) small group learning;
Length/frequency	1) 30 hours face-to-face intensive EBM education during orientation; 2) longitudinal: unclear length & frequency	2 weeks (3 half-day clinics per week, with the remainder of the time available for EBM)	5 x 1-hour monthly research and statistics lectures	4 x 1 hour weekly sessions	Monthly conference
Teachers/ facilitators	Faculty, residents	Faculty, medical librarian	Research program coordinator	Teacher with expertise in epidemiology and biostatistics	Librarians, pharmacists, others
Clinically integrated (lectures versus point of care)	Yes	Yes	No	No	No

Curriculum content	1) Introduction to Information Mastery; 2) group exercise: is It a POEM; 3) don't panic: statistics you can understand; 4) power reading of journals; 5) expert- based information delivery systems; 6) diagnostic testing: Bayes' Theorem, evaluating studies of diagnostic tests; 7) evaluation of Grand Rounds using the CME evaluation form; 8) obtaining useful information from pharmaceutical representatives; 9) using Clinical Decision Rules; 10) clinical Jazz: harmonizing clinical experience with Evidence-based Medicine	Critical evaluation of articles about diagnosis, therapy, prognosis, meta- analysis and decision making, and how to ask clinical questions. PICO, formulate questions, generate searches, developing searching strategies, high quality EBM resources, information mastery; essential EBM concepts, including clinical question development, levels of evidence search strategies, and appraisal techniques; critically appraised topics - each CAT is structured to include the 'clinical bottom line' answer to the question	Hypothesis testing, variable types, study designs, clinical trial phases, bias and confounding, odds and risk ratios, sensitivity and specificity, 2 x 2 tables, incidence and prevalence, representative descriptive statistic, standard deviation and measures of variability, multiple comparisons, types of statistical tests, power and sample size calculations, number needed to harm/treat, confounding assessment and adjustment, patient safety and quality improvement methods, national surgical quality improvement program, surgical care improvement project measures	1) hypothesis testing and exploratory data analysis; 2) confirmatory data analysis; 3) study designs; 4) each resident had to prepare a hypothetical study in advance which would answer a clinical or research question	1) Introduction and PICO questions; 2) live literature search and analysis of the results; 3) article analysis focusing on assigned key concepts of analyzing different types of articles such as therapy, diagnosis, etc., so that each month focused on a different concept in depth; and 4) article application to patient population; 5) modified journal club format.
Curriculum development process described	Yes, Kerns six step approach used	Not discussed	Not discussed	Yes, Kerns six step approach used	Yes, Kerns six step approach used

Outcome measures	Mastery of residents' evidence-based medicine knowledge and skills as well as their confidence at critically appraising medical literature and using evidence to inform clinical decisions.	Level of confidence in basic EBM skills	Improvement in research and statistics ABSITE scores	Assessment of resident performance, assessment of curriculum	Impact that the curriculum changes (including librarian involvement) had on the residents' perceived abilities to integrate knowledge gained from the EBM conference into meaningful literature searches.

Evaluation method	Fresno Test of Evidence-based Medicine; an attitude questionnaire at the start of the curriculum and then again before graduation	self-assessment of EBM skills (interns) - adapted from Fresno test	ABSITE examinations	20-item multiple-choice knowledge test; written survey about the curriculum	survey
					
Results	Modified Fresno Test scores significantly improved from 104.0 to 121.5. Using a pass/fail approach, nine residents (40.1%) passed the test at the start of training, increasing to 17 (73.4%) at the end of the intervention. Confidence in critical appraisal scores increased from an average 17.90 (95% CI=16.55–19.25) to 21.10 (95% CI=19.49–22.71), out of a possible score of 25. Attitudes regarding confidence in the use of evidence and a decreased reliance on experts were also improved following the curriculum.	Interns significantly increased their confidence over the course of the rotation; scores improved postrotation in all 3 areas tested: EBM terms and concepts 81% to 97%; quantitative skills 51% to 80%; question formulation and searching 71% to 92%, with the total score increasing from 63% to 87%; residents reported applying the EBM skills they learned to patient care (86%) and that these skills were reinforced in the teaching they received outside of the rotation (81%); all residents felt that the EBM curriculum had improved patient care.	Residents demonstrated significant improvement in postcurriculum examination scores for research and statistics items, correct responses increased 27% (p< .001), residents were 5 times more likely to achieve a perfect score on research and statistics items postcurriculum (p< .001).	Mean±SD proportion of correct answers for the 52 course participants was 58±16%; historical controls – 26 residents in the same programme and 277 residents from other residency programmes – scored less well (48±14%, p=0.01 and 41±15%, p<0.0001, respectively).	Overall, residents found the optional technology session helpful, appreciated librarian involvement during EBM conferences, increased their knowledge of library resources, reported improved knowledge and comfort using electronic library resources after the curriculum changes were implemented, and felt that they could integrate knowledge learned during the EBM conference series into meaningful literature searches.

**Table 3:** Description of abstracts included in the review of research on residents' evidence-based medicine curricula

Author (year)		Al Jalbout et al. (2019)		Gupta et al. (2014)		Kao et al. (2012)		Kluesner (2015)	Merritt (2015)
Study design		Pre-post design		Prospective cohort		Pre-post study		Pre-post study	Pre-post study
Country		USA		USA		USA		USA	USA
Specialty		Emergency Medicine		Internal Medicine		Internal Medicine		not reported	Internal Medicine
Number of Participants, n		14		not reported		11		26	108 (10-12/session)
Study period		16 months		Not reported		1 year		2 years	6 months
	
EBM Curriculum Structure		8 structured interactive online modules and a librarian meeting		5 modules: 1) introduction to EBM; 2) randomized control trials; 3) cohort and case-control studies 4) diagnostic tests. 5) meta-analyses		2 week block rotation for first and second year residents		Journal Club integrated with EBM principles	Interactive lectures and case discussion
Length/frequency		2 weeks		Not reported		2 weeks		18 months	Once a month for 30 minutes
Teachers/ facilitators		Librarian		Physician, EBM expert, instructional designer		Internal medicine faculty, librarians		Teaching resident, core EBM faculty	Not reported
		
Curriculum content		Process of EMB, study design, validity, and biostatics, PICO and search strategies		1) introduction to EBM. 2) randomized control trials. 3) cohort and case control studies. 4) diagnostic tests. 5) meta-analyses		Hands-on sessions to teach literature search and critical appraisal		1) peer instruction model and peer to peer discussion coordinated by a teaching resident, 2) dedicated EBM lecture delivered at the begining of each journal club, 3) identification of	1) assessment of risks and benefits of treatment. 2) rational use of diagnostic testing
	
Outcome measures		EBM knowledge and skills		Satisfaction with the course, milestones, correctness of responses, number of attempts, need for remediation		EBM skills and knowledge		EBM skills and knowledge	Comfort in understanding of material

Evaluation method		Fresno test		survey		Fresno test; Resident self assessed comfort with EBM resources		Fresno test administered at the end of each year	comfort via 5pt Likert scale; critical risk interpretation test (CRIT)
Results		1) Fresno median pre-test 80, median post-test 103 (p=0.002). 2) PICO question (p=0.009), internal validity (p=0.018), study design (p=0.004); no significant improvement among search strategy or biostatistics knowledge		97% of residents were satisfied or very satisfied with the course		1) Fresno mean pre-test 114.3, mean post-test 154.6 (p<0.001). 2) Likert scale (p<0.001) for: searching and using MeSH terms, advanced limits, subheadings in Ovid, and conducting and presenting a literature review		Fresno pretest 110.16, year 1 posttest 127.82, year 2 posttest 127.07, (p=0.011)	1) "furthered understanding of how to practice EBM" 4.6/5 of Likert. 2) "use skills learned in these sessions during regular practice" 4.5/5 on Likert

Author (year)		Moll (2012)		Mroz and Carroll (2019)		Schwartz et al. (2000)		Vesbianu and Rodriguez (2018)	Vogel (2000)
Study design		Pre-post study		Prospective cohort		Pre-post study		Pre-post study	Pre-post study
Country		USA		USA		USA		USA	USA
Specialty		Emergency Medicine		Internal Medicine		Primary Care		internal medicine	internal medicine
Number of Participants, n		not specified		68		14		30	42
Study period		3 years		1 year		6 weeks		6 months	One year

EBM Curriculum Structure		Modules		Interactive lectures and problem based learning		Self-reported critical appraisal on 13 items, and self-reported electronic searching skills on 17 items		Small group ambulatory didactics	Didactic, with residents giving presentations at the end of the course
Length/frequency		Four 6-month modules		Six 1-hour sessions over one year		60 hours over 6 weeks		1 hour sessions every week	8 weeks
Teachers/ facilitators		Faculty		Not reported		Not reported		Faculty	Unclear

Curriculum content		1) introduction and tools needed; 2) small groups formulate a clinical question, research and rate literature; 3) implementation module, knowledge translation and set		1) recognition of overconfidence and understanding probability; 2) test interpretation; 3) study design; 4) asking a clinical question; 5) abstract review; 6) feedback and skills		1) critical appraisal 2) electronic searching skills		1) appraise and understand medical literature. 2) incorporate learned EBM knowledge into patient care	1) developing clinical questions from patient encounters 2) finding best- quality information sources 3) critical appraisal

Outcome measures		EBM sources, knowledge translation tool use and retention		EBM skills and knowledge; opinions on the new curriculum		Attitudes towards EBM curriculum		Level of confidence	Survey of EBM attitudes and abilities, computer searching skills, opinions on curriculum, confidence in skills learned

Evaluation method		self-reported questionnaire		Fresno, resident evaluation		survey, 6-item 4pt nihilism scale to address distrust of research methods and applicability of research articles		self-reported EBM skills questionnaire	self-reports, surveys, and written tests
Results		EBM sources are the first query in 67% compared to 50% at the start, and 63% self-identify a change in how they obtain information		1) Fresno post-test 124; 2) resident course rating 7.8 (SD 1.8) on a 10 point Likert scale		1) critical appraisal before study 71%, after study 85%, (p=0.002). 2) electronic searching skills before study 68%, after 83%, (p=0.001).		perceived level of confidence improved from 3.62 to 3.89, (p=0.05)	1st written pretest 49% posttest 86%, 2nd written pretest 42% posttest 57%. (p<0.001) for each

Author (year)	Vom Eigen (2002)		Vu et al. (2017)		Walkey and Fairchild (2006)		Zipkin et al. (2010)		
Study design	Pre-post study		Pre-post study		Pre-post study		Pre-post study		
Country	USA		USA		USA		USA		
Specialty	Primary Care Internal Medicine		Pediatric		primary care		Internal Medicine		
Number of Participants, n	46		23		21		18		
Study period	3 years		1 year		Not reported		Not reported		

EBM Curriculum Structure	interactive didactic small groups		Small groups, didactics		Didactic, interactive		Small, interactive groups		
Length/frequency	10-12 weekly sessions for 1 hour over the course of the 3 month curriculum		Monthly conferences		1.5 hr lecture on didactics followed by 15 min residents presentation on		4 half-day sessions over one week		
Teachers/ facilitators	Instructor		Residents and faculty		More senior residents residents		Not reported		

Curriculum content	Biostatics, informatics, clinical epidemiology, patient care issues, search for relevant research articles, discussion lead by instructor		Formulate a clinical question, selected and appraised a single article, present an overview, and facilitated discussions		1) didactic session, sample patient encounters. 2) patient contact, generating and question and presentation based on encounter. 3) opportunity to teach other residents		Understanding study design, bias and random error, diagnostics, screening, treatments, harm, and prognostics		

Outcome measures	Pre/post test of knowledge as well as self-evaluated changes in clinical knowledge, skills, practice, and ability to evaluate medical literature		level of EBM education, ability to appraise articles on Likert scale		Ability to form clinical questions		Knowledge, quality of education, written feedback		

Evaluation method	38 item test		Berlin Questionnaire and Assessing Competency in EBM (ACE)		adapted Fresno test (72 pt scale)		25 point multiple choice test		
Results	mean score of pre and post tests improved from 65.3% to 73.7% (p<0. 001).		1) Berlin Questionnaire pretest 63%, posttest 76%, (p<0.001). 2) ACE pretest 63%, posttest 67%, (p=0.27). 3) 2.9/5 Likert for quality of education in EBM during medical school. Rated ability to appraise articles pretest was 2.8 and posttest was 3.2 on Likert (p=0.03)		1) Fresno pre/post scores increased by 55% (p<0.0001). 2) key subsections: ability to identify EBM sources (120% increase in correctness, p<0.0001) and ability to form questions in PICO format (36% increase, p=0.03)		pretest mean 14.17 (SD 4), posttest mean 17.11 (2.4), (p=0.008)		
									

Eighteen studies had a moderate risk of bias, and six studies had a low risk of bias. After applying the NOS criteria, 2 of the four observational prospective cohort studies were assessed as having a low risk of bias^38^^,^^39^ and the other two studies were assessed as having a moderate risk of bias.^40^^,^^41^ From the randomized controlled trials, Kim and colleagues^42^ had a low risk of bias, and Kortekaas and colleagues^43^ had a moderate risk of bias. From the 18 pre-post studies, only 3 of them were assessed as being at low risk of bias;^44^^-^^46^ the rest were considered at moderate risk of bias. None of the studies were evaluated as having a high risk of bias.

### Synthesis of results

Details regarding the EBM curriculum structure, length, frequency, teachers, content, outcome measures, evaluation method, and results can be found in Table 2 for the full-text articles and in Table 3 for the abstracts.

### EBM curriculum structure

The EBM curricular structure was found to be highly variable from study to study. EBM was taught through multiple venues: workshops,^37^^,^^38^^,^^40^^,^^41^^,^^47^ journal club format,^10^^,^^29^^,^^30^^,^^32^^,^^33^^,^^4^ resident-led sessions,^10^^,^^31^^,^^40^^,^^44^^,^^49^^-^^51^ didactic lectures,^28^^,^^39^ librarian sessions^10^^,^^33^^,^^50^ EBM teaching rounds,^44^^,^^52^^,^^53^ online sessions,^47^^,^^52^ hands-on interactive sessions,^33^^,^^36^^,^^38^ small group sessions,^4^^,^^10^^,^^26^^,^^37^^,^^49^^,^^50^^,^^54^ multiple choice questions,^28^ modules,^24^^,^^35^ self-paced modules,^45^ one-on-one meetings,^10^^,^^55^ morning report,^44^ EBM rotation,^47^ point-of-care teaching,^33^ block rotation,^56^ and problem based learning.^57^  The curricular structure has been reported to be developed on the adult learning theory principles in 3 articles.^10^^,^^46^^,^^58^ Most curricula contained more than 2 teaching methods, with only 9 curricula reporting on a single intervention repeated for multiple times: interactive workshops,^59^ EBM teaching rounds,^53^ Journal Club,^29^^,^^60^ one-on-one meetings,^55^ self-paced modules,^45^ modules,^24^ and small group sessions.^26^^,^^36^^,^^54^

### EBM curriculum length / frequency / teachers

The majority of the EBM curricula was longitudinal with different lengths, from weekly 1-hour sessions for 4 weeks^49^ to 3 year curriculum.^32^ Some curricula were reported in blocks of 2 weeks^25^^,^^56^ 7 weeks,^46^ 8 weeks,^38^ two 2-hour sessions,^13^ daily EBM teaching rounds for one month,^53^ or intensive 30 hours of EBM training during a month.^33^ The frequency of the sessions in each curriculum was variable. In the longitudinal curricula, the sessions were as often as weekly,^34^^,^^49^ monthly,^29^^,^^60^ or a few months every year.^41^ Only two curricula were designed based on the PGY level, reporting different sessions and activities based on the year of training.^4^^,^^32^ The rest of the curricula were designed for the generalized audience of all physicians in training. There was a multitude of EBM teachers involved in curriculum delivery in most of the studies, faculty physician, faculty librarian, all level residents, learning coach,^55^ research program coordinator,^28^ nonphysician biostatistician, research assistant^32^ and pharmacist.^50^ Four out of the 29 full-text articles and 5 out of the 14 abstracts didn't specify their EBM curriculum teachers.

### EBM curriculum content

The majority of the reported EBM curricula address all five of the EBM competencies.^1^ Bentley and colleagues^45^ Konen and colleagues^32^ and Schwartz and colleagues^61^ had the main focus on critical appraisal skills. Kitchens and colleagues^34^ used a series of articles as a basis for critical appraisal teaching. Trickey and colleagues^28^ and Windish and colleagues^49^ had a major focus on teaching basic and advanced biostatistics.

### EBM curriculum development process

Kern's six-step approach to design an experiential curriculum in knowledge translation has been a well-established model for the curriculum development process that has been shown to lead to successful implementation and long-term sustainability.^62^^,^^63^ The included steps are: 1. problem identification and general needs assessment; 2. needs assessment of targeted learners; 3. goals and objectives; 4. educational strategies; 5. implementation; 6. evaluation and feedback. From the 29 full-text articles, only 5 of them (17%) mentioned that they used Kern's six-step approach for curriculum development.^33^^,^^49^^,^^50^^,^^52^^,^^59^ Allan and colleagues and Green and colleagues reported on all the steps as well but without mentioning on the approach.^46^^,^^48^ George and colleagues reported on all the steps except goals and objectives.^55^ Nicholson and colleagues reported on all the steps without the needs assessment.^53^

### Clinical integration of EBM curriculum

We defined clinical integration of the EBM curriculum if the EBM principles, steps, or concepts were taught in the context of patient care. The EBM curriculum was clinically integrated into 29.3% (10 out of 29) of the full-text articles. Ten percent of the EBM curricula were only partially clinically integrated^4^^,^^43^^,^^44^, and the rest, which was the vast majority, were not clinically integrated.

### Outcome measures

Most of the EBM curricula reported on level 2 (EBM skills and knowledge), 45% (13 out of the 29 full-text articles) and 57% (8 out of the 14 abstracts). Fewer articles reported on level 1 (attitude toward EBM and participants' satisfaction): 17% (5 out of 29 full-text studies) and 35.7% (5 out of 14 abstracts). A total of 10 articles (9 full texts and one abstract) reported on both level 1 and level 2. We found 2 full-text articles that also reported on level 3 (behavior, the degree to which participants apply what they learned during training when they are back on the job). Kortekaas and colleagues^43^ reported on information-seeking behavior, and Ross and colleagues^59^ reported on EBM terminology usage on tape-recorded interactions.

### Evaluation method

From the 29 full-text articles, only 4 used the validated Fresno^33^^,^^42^^,^^44^^,^^60^ and 1 used the validated Berlin questionnaire^45^ to assess outcomes. Out of the 14 abstracts, 5 used the validated Fresno^25^^,^^31^^,^^36^^,^^56^^,^^57^ and 1 used the validated Berlin questionnaire.^26^ Nelson and colleagues^58^ reported the usage of a 20 question previously validated questionnaire. The rest of the studies used unvalidated surveys and questionnaires to assess their outcomes. Eighteen studies out of 43 (41.8%) reported on the usage of surveys to assess the residents' attitudes and comfort with EBM concepts, and 24 out of 43 studies (55.8%) used some questionnaire (multiple choice examinations) to assess the EBM knowledge and skills. Only three studies (7%) also used qualitative analysis (focus group) to assess the residents' reaction to the EBM curriculum implementation.^4^^,^^44^^,^^55^

### Barriers to EBM curriculum implementation

Barriers reported during the EBM curriculum implementation were categorized into three main subsets: barriers to teach, barriers to learning, and barriers to practice EBM. (Table 4) Most commonly, EBM curriculum articles reported on barriers to teach, with time and difficulty to recruit teachers being the most frequent barriers reported. Time has been as well reported as the main barrier to practice EBM.

**Table 4:** Barriers to EBM curriculum implementation

Barriers to teach	
	- Learners not completing pre-reading^48^
	- Lack of dedicated teaching time^13,44,46,60^
	- Learner fatigue with repetition^52^
	- Varying resident abilities/knowledge^40^
	- Difficult to recruit teachers^32,46,53,60^
	- Lack of consensus on teaching strategies/curriculum topics^13^
Barriers to learning	
	- Learner fatigue with repetition^52^
Barriers to practice	
	- Time restraints^10,27,48,50^
	- Lack of knowledge^10,48^
	- Lack of access to resources^48,50^
	- Subjects not taught frequently enough^38^
	- Poor search skills and database knowledge^13,42,5^

### Does competency in EBM improve after EBM curriculum implementation?

The heterogeneity of the studies interventions and outcome assessments precludes any attempt to combine the results quantitatively. All the published studies and abstracts, independent of the EBM curriculum structure and the evaluation method used, found an improvement in the residents' attitudes and/or EBM skills and knowledge. Bentley and colleagues^45^ reported a 33.4% increase in the Berlin questionnaire posttest. George and colleagues^55^ also reported a significant increase of 31% in the post quiz score. Kenefick and colleagues^13^ showed a 58% improvement in the posttest regarding PICO question and 53% improvement in the searching skills. Al Jabout and colleagues^25^ reported a 23point improvement in the median Fresno posttest, which was statistically significant at p=0.002. Gupta and colleagues^35^ had 97% of residents satisfied with the EBM curriculum. There was a 37% improvement in the written posttest in the study by Vogel and colleagues^51^ and Walkey and colleagues^36^ had an impressive improvement in the Fresno test score of 55%. None of the studies reported on long term, post-residency training, retention of EBM skills, or knowledge.

## Discussion

### Summary of evidence

We reviewed a total of 43 articles, from which 29 full-text articles and 14 abstracts reporting on the EBM curriculum. Overall the body of evidence regarding EBM curriculum structure, efficacy, and barriers is small and of limited quality. The studies included were considerably heterogeneous with respect to their EBM curriculum structure, content, length, evaluation method, and outcome assessment. A common finding within the studies was that multiple teaching strategies were implemented simultaneously. All studies showed an improvement in educational outcomes across different EBM curricula. However, no specific curriculum was found to be superior because there was no direct comparison.

The curricular structure in each article included was described in adequate detail that would allow reproduction and generalizability. This is in contrast with what Green and colleagues^64^ noticed in their systematic review of EBM curricula, that many of the reports suffer from the incomplete description of the curricula. The vast majority of the curricula contain multiple strategies to teach EBM in multiple settings. The most common integral session in the EBM curricula reported was journal club, followed by small group sessions, resident-led sessions, hands-on interactive sessions, and workshops. Also, the majority of the EBM curricula reported has been comprehensive, addressing all of the EBM competencies. It is known that offering multiple EBM interventions in multiple learning venues throughout residency training will facilitate the reinforcement and retention of key skills and behaviors.^10^ Some suggest adopting a spiral approach to EBM teaching, whereby concepts increase in complexity and are reinforced throughout learning experiences.^65^

Moving EBM instruction out of the classroom and into the clinical arena is crucial for the application.^4^ When residents have the time and opportunity to integrate EBM competencies during real-time patient care, they shift from a paternalistic to a participatory decision-making style.^53^ This goes along with the adult-learning theory principles and should be considered in future EBM curriculum development. In his review, Coomarasamy and colleagues^66^ reported that only integrating EBM teaching with clinical activities was associated with increased EBM competency across all levels (attitudes, knowledge, skills, behaviors) in medical postgraduates.

Our findings are consistent with Green and colleagues^52^ regarding lack of attention to curriculum development principles, as only less than a third of the full-text articles reported on the curriculum development and implementation steps. Eight of the 9 studies that explained the EBM curriculum development and implementation process were published in the last 20 years, which can suggest the hypothesis that in the previous 20 years, with the progression of science, the introduction of reporting guidelines and also with the wide availability of the internet, scientists improved their knowledge in curriculum development and reporting. The lack of attention to curriculum development principles could be explained by the lack of knowledge and training in curriculum development of the curriculum developers.

Very few studies reported on the Kirkpatrick' evaluation model level 3, behavioral change, and no studies reported on level 4, results/patient outcomes. These findings are similar to previous systematic reviews data on medical education interventions.^46^ This could be due to the fact that assessing level 3 and level 4 has been previously described as difficult to achieve because it is significantly more time-consuming and requires a more demanding evaluation. Level 4 should be viewed as the primary goal of any training program, but it is very challenging to assess whether certain patient outcomes can be linked to the EBM curriculum intervention or not. In a systematic review of 599 research articles published in three major medical education journals, patient outcomes accounted for only 0.7% of all articles.^67^

Ilic and colleagues^7^ describe criticism of the current state of the evidence in this field as being the lack of a uniform, validated assessment tool that can measure all aspects of the EBM competencies. Our findings are similar, with the vast majority of curricula being evaluated by non-validated tools. A 2006 systematic review identified 104 unique instruments with reasonable validity for evaluating some domains of evidence-based practice and may be targeted to different evaluation needs.^68^ The majority (90%) were not high-quality instruments with established inter-rater reliability, objective outcome measures, and three or more types of established validity. To this day, only the Fresno test of competence in evidence-based medicine^69^ and the Berlin Questionnaire^70^ are high-quality instruments identified as evaluating EBM knowledge and skills across at least 3 of the 5 EBM steps.

Time has been mentioned as the most frequent barrier to teach and practice EBM. This finding is consistent with prior literature. Dijk and colleagues^17^ reported in his systematic review on barriers to residents' practicing EBM that limited available time has been the most often mentioned and primary barrier for residents. Another important barrier that has been very frequently reported was the lack of EBM experts/teachers. This barrier has significant implications in the successful implementation of any EBM curriculum and constitutes one of the first steps in curriculum development.

### Strengths and limitations

The strengths of our scopic review include systematic reproducible search of the literature, explicit methods which limit bias, reliable and accurate conclusions and the ability to bring a vast body of literature to an update review. One of the main limitations of this review is the likely publication bias. Because negative studies are less likely to be written, submitted or published, especially in medical education, the studies' results might be skewed toward reporting the positive effects of the interventions. We also noticed that the published data on EBM curriculum is geographically limited and thought that it might be due to publication bias and might further bias the results by not fully representing the EBM curricula from many other countries. It is also possible that there are many innovative and effective EBM curricula that have never been reported. Another major limitation is the quality of the study design of the full manuscripts included. The heterogeneity of the interventions and outcomes constitutes a limitation in the ability to draw a conclusion based on this data. One other limitation worth noting is the small sample size in the majority of these studies, as a convenience sample was most often used, and the studies were single centre—none of the studies reported on patient care impact assessment.

## Conclusions

This is the first scoping review to answer the question regarding the structure of EBM curricula and barriers found during the EBM curriculum implementation process. EBM competencies are necessary for providing high-quality patient care within physicians in training education. The current body of literature available to guide educators in EBM curriculum development and barriers to implement and teach EBM is enough to constitute a strong scaffold for developing any EBM curriculum. Teaching EBM concepts can use any method and can be integrated into almost any setting. Special attention should be paid to the potential barriers during the curriculum implementation, and ways to overcome them should be identified early in the curriculum development process. Given the amount of time and resources needed to develop and implement an EBM curriculum, it is imperative to follow the curriculum development steps and use validated assessment tools. During that development, medical educators should design rigorous evaluation strategies with more meaningful clinical outcomes that would evaluate Kirkpatrick's learning levels 3 and 4. Further studies should focus on studying long term retention of the EBM concepts, skills and knowledge as they remain unknown for now.

### Conflict of Interest

The authors declare that they have no conflict of interest.

## Supplementary material

Supplementary file 1 Appendix 1. Quality of Pre/Post studies

Supplementary file 2 Appendix 2. Quality of prospective cohort studies and randomized controlled trials
