# Supplementary material for: Evidence-based medicine curricula and barriers for physicians in training: a scoping review
Source: Int J Med Educ. 2021 May 28;12:101–24. doi: 10.5116/ijme.6097.ccc0 (PMC8411338; doi:10.5116/ijme.6097.ccc0)
Supplement: Supplementary file 1 — Appendix 1. Quality of Pre/Post studies [file ijme-12-101-S1.pdf]

Appendix 1.

Quality of Pre/Post studies

Author (year)	1	2	3	4	5	6	7	8	9	10	11	12	Score
Allan et al. (2008)	+	+	+	-	-	+	+	+	-	+	-	-	M
Aneese et al. (2019)	+	+	+	-	-	+	+	+	+	+	-	+	L
Bentley et al. (2018)	+	+	+	+	-	+	+	CD	+	+	-	+	L
Chitkara et al. (2016)	+	+	+	CD	-	+	+	CD	-	+	-	+	M
Friedman et al. (2010)	+	+	+	-	-	+	+	-	-	+	-	-	M
George et al. (2010)	+	+	+	+	-	+	+	-	+	+	-	-	M
Green and Ellis (1997)	+	+	+	+	-	+	+	+	+	+	-	-	L
Halalau et al (2016)	+	+	+	-	-	+	-	-	+	+	-	-	M
Kenefick et al. (2013)	+	-	+	CD	-	+	-	-	+	+	-	-	M
Kitchens and Pfeifer (1989)	+	+	+	+	-	+	+	-	+	+	-	-	M
Lentscher and Batig (2017)	+	+	+	+	-	+	-	-	-	+	-	-	M
Mohr et al. (2015)	+	+	+	+	-	+	+	CD	-	+	-	-	M
Nelson et al. (2017)	+	+	+	+	-	+	+	CD	+	+	-	-	M
Ross and Verdieck (2003)	+	+	+	-	-	+	+	-	+	+	-	-	M
Shaughnessy et al. (2012)	+	+	+	+	-	+	+	-	+	+	-	-	M
Thom et al. (2004)	+	+	+	CD	-	+	+	CD	+	+	-	-	M
Trickey et al. (2014)	+	+	+	-	-	+	+	CD	-	+	-	-	M
Windish (2011)	+	+	+	+	-	+	+	CD	+	+	-	-	M
