# Supplementary material for: Evidence-based medicine curricula and barriers for physicians in training: a scoping review
Source: Int J Med Educ. 2021 May 28;12:101–24. doi: 10.5116/ijme.6097.ccc0 (PMC8411338; doi:10.5116/ijme.6097.ccc0)
Supplement: Supplementary file 2 — Appendix 2. Quality of prospective cohort studies and randomized controlled trials [file ijme-12-101-S2.pdf]

Appendix 2.

Quality of prospective cohort studies and randomized controlled trials

Prospective cohort studies

Author (year)	Representativeness of the exposed cohort	Selection of the non-exposed cohort	Ascertainment of exposure	Demonstration that outcome of interest was not present at start of study	Compatibility of cohorts on the basis of the design or analysis	Assessment of outcome	Was follow-up long enough for outcomes to occur	Adequacy of follow up of cohorts	Overall risk of bias
Gehlbach et al. (1980)	+	-	+	-	+	+	+	+	Moderate
Keddis et al. (2011)	+	+	+	-	+	+	+	-	Low
Kohlwes et al. (2006)	+	+	+	-	+	+	+	+	Low
Luciano et al. (2016)	+	-	+	-	+	+	+	+	Moderate

Randomized controlled trials

Author (year)	Randomization	Allocation concealment	Selective reporting	Other sources of bias	Blinding (participants, personnel)	Blinding, outcome assessment	Incomplete outcome data?
Kim et al. (2008)	Low	Low	Low	High	High	Low	Low
Kortekaas et al. (2016)	Low	Low	Low	High	High	Unclear	High

+: fulfilled criteria, -: did not fulfill criteria; Risk of bias: high, low, unclear
